# Mental disorders and related factors in higher education professors working remotely: a correlational study

**DOI:** 10.47626/1679-4435-2021-937

**Published:** 2024-02-16

**Authors:** Pérola Liciane Baptista Cruz-e-Silva, Renata Lourdes Rodrigues Caldeira, Sameque Kaique Batista de Sá, Kaio Patrick de Paula, Ellen Raquech Torquatro Branco, Sidalia Araujo Vitor da Silva

**Affiliations:** 1 Nursing, Faculdades Integradas de Jahu, Jaú, SP, Brazil

**Keywords:** mental disorders, faculty, occupational health, universities, COVID-19, transtornos mentais, docentes, saúde do trabalhador, educação superior, COVID-19

## Abstract

**Introduction:**

Recent changes in labor relations and the employment market in university education have posed significant challenges and increased workloads for faculty a situation that has been exacerbated by the global epidemiological status in 2020.

**Objectives:**

To investigate the presence of mental disorders and associated factors in faculty members at a private university in São Paulo, Brazil.

**Methods:**

This is a quantitative, descriptive, exploratory cross-sectional study conducted in June 2021, using online questionnaires to collect social and demographic and lifestyle data, namely, Self-Reporting Questionnaire (SRQ-20) and the Patient Health Questionnaire (PHQ-9). The presence of associations between variables was investigated using odds ratio and relative risk.

**Results:**

Participants included 49 professors, mostly women, aged between 29-78. Among the subjects, 61.2% had a body mass index above the recommended level and 40.8% were considered sedentary. A chronic illness was reported by 40.8% and 65.3% reported continued-use medication. A common mental disorder was reported by 28.5%, and the same prevalence was found for depression, with a positive association with being considered sedentary, overweight or obese, drinking alcohol at least once a week, and having worked as a professor for less than 5 years.

**Conclusions:**

The study showed a significant level of emotional distress among professors, associated with lifestyle habits that can compromise their quality of life and professional performance.

## Abstract (pt)

**Introdução:**

Recentes mudanças nas relações e mercado de trabalho no setor da educação superior trazem desafios e sobrecargas importantes aos corpos docentes, o que, pela emergência da situação epidemiológica mundial, foi agravado no ano de 2020.

**Objetivos:**

Investigar a presença de transtornos mentais e fatores associados em docentes do ensino superior em instituição privada do interior do estado de São Paulo.

**Métodos:**

Pesquisa quantitativa, descritiva exploratória, de corte transversal, transcorrida em junho de 2021, utilizando-se de instrumentos por meio digital contendo dados sociodemográficos e de hábitos de vida: o Self Reporting Questionnaire e o Patient Health Questionnaire. Investigada a presença de associações entre variáveis por meio da razão de chance *(odds ratio)* e do risco relativo.

**Resultados:**

Participaram 49 docentes, em sua maioria do sexo feminino, entre os 29 e 78 anos. Em 61,2% dos indivíduos, o índice de massa corporal esteve acima do recomendado e, ainda, 40,8% foram pontuados como sedentários. A presença de doença crônica foi relatada por 40,8%, e o uso contínuo de medicamentos por 65,3%. A presença de transtorno mental comum esteve presente em 28,5%, sendo a mesma prevalência encontrada de quadros depressivos, apresentando associação positiva com o reconhecer-se sedentário, sobrepeso ou obesidade, consumo de bebida alcoólica pelo menos uma vez na semana e tempo de trabalho docente menor que 5 anos.

**Conclusões:**

O estudo apontou um quadro relevante de sofrimentos mentais entre os docentes, associados a hábitos de vida, que podem comprometer a qualidade de vida e de trabalho.

## INTRODUCTION

Brazil's Ministry of Health (Ministério da Educação) Employee Assistance Handbook^1^ (Caderno de Atenção aos Trabalhadores) recognizes that work and their relations are an important determinant of the health and illness process of employees and their families. Not only is it defined as a protective factor for health conditions, but it also favors the formation of a support network and social inclusion, as well as the generation of income; on the other hand, work-related health problems are not uncommon and can have a significant impact on the quality of life of employees.^1^

In this context, contemporary transformations in various social dimensions, partly fueled by technological and communication developments, have exposed employees in different fields to hyperconnected environments, with demands for high productivity, consumerism, and individualism, directly interfering in their human relationships, values, and quality of life.^2^

The pressure to solve problems in very short deadlines, continuous training, and almost full-time focus on work, reinforced by requests sent in by the general public, institutions, and access control devices, are also experienced by a number of professionals, including professors.^2,3^

Especially in universities, where professors are required to be committed to activities such as teaching, research, and further education - the "academic triad" - the fast-paced production of knowledge, the welcoming of students, and administrative and educational control tasks are situations that accumulate in their routine, significantly affecting their quality of life, especially their social and emotional quality of life.^3^

In a study with elementary and high school teachers in Paraná, Brazil, 29.73% of the participants reported suffering from mental illness and 23.98% from musculoskeletal disorders. When screening for minor mental disorders (MMD), indicative scores were found in 75.27% of the interviewees and, when screening for depressive symptoms, 44.04% of the interviewees reported them.^4^

This same study reported that 23.72% of teachers were absent from work due to emotional distress and 32.31% used psychoactive medication. In addition, a positive association with these disorders was described among professors who worked on assignments they took home, a higher number of classes per professor, and had worked for more than 20 years.^4^

Common mental disorders (CMDs) or MMDs deal with emotional distress associated with psychosomatic, depressive, insomnia, irritability, forgetfulness, and anxiety symptoms, negatively affecting quality of life and public health, with a prevalence of around 20% in the general population, although not included in the diagnostic criteria of the classic psychiatric syndromes.^5,6^

In a study including faculty members at a public university in Bahia, Brazil, the overall CMD rate was 29.9%, with a positive association with feelings of work overload, perceived pressure to publish, inadequate classroom conditions, and dissatisfaction with the university.^3^

In 2020, a new global scenario experienced as a result of the COVID-19 pandemic brings new elements that have contributed to transformations in working conditions. In response to the social isolation measures widely encouraged by international bodies, exceptional regulations from the Ministry of Education, through Ordinance No. 345/2020, replace face-to-face teaching in ongoing courses with remote classes.^7^ As a result, new challenges related to handling technology and communicating with distance learners were introduced in an effort to finish the school year.^8^ This scenario will be maintained in the first half of 2021, given the upsurge in the epidemiological situation of COVID-19.

Beyond the education field, this new scenario has had implications for the economy, with greater job instability, unemployment, informality, and an increase in social vulnerabilities. Some sectors traditionally focused on collective activities, such as education, culture and leisure, have been impacted more intensely by isolation measures.^9^

The effort to adapt to new work contexts has led to fragmentation and isolation of teaching tasks, with a potential loss in the quality of education, professional frustration, and illness. These conditions have an effect on external relationships, family members, social networks, and overall quality of life.^2^

In this regard, the difficulties in achieving the new objectives in this working context, associated with the handling of technologies and the need to change teaching methods to models that allow and encourage interactivity and dialogical reflection in the digital environment, have shown increased pressure among professors and work-related illness, especially in the psychological and social field.^8^

This research therefore sets out to answer the following leading question: after one year of working remotely, how do university professors feel about their mental health and quality of life?

The aim is to investigate the presence of mental disorders and their associated causes among university professors at a private university in the inland region of São Paulo, Brazil.

## METHODS

This is a quantitative, descriptive, exploratory, cross-sectional study conducted at a university located in the inland of São Paulo, Brazil.

The population includes professors who are currently working and who taught undergraduate courses in 2020. All professors the university indicated were invited to participate voluntarily by electronic means. The inclusion criteria were professors who continued to perform their tasks remotely with students during the period when face-to-face classes were suspended in 2020.

The exclusion criteria were having been hired as a professor at the university later than August 2020, having been on sick leave for more than 30 days in 2020, or having stopped teaching the courses and not continuing to teach the subject remotely. The professors signed an informed consent form and agreed to participate in the survey electronically, using Google Forms.

Data were collected in June 2021 using a structured digital questionnaire containing social and demographic data and data on lifestyle and health habits. After the initial questionnaire, the Self Reporting Questionnaire (SRQ-20), a tool validated in Brazil for CMD screening in primary health care services, was applied.^10^ The cutoff point was different for men and women, with six or more affirmative answers for the former and eight or more for the latter. Google Forms was used to apply the questionnaires.

The Patient Health Questionnaire (PHQ-9), validated in Brazil for simple screening of depressive symptoms, was the third tool used. To analyze the results, we considered the sum of the answers in a continuous manner, with a cutoff point > 9, and using the algorithm the authors recommend. The following were also considered positive: the existence of five or more symptoms, scored between 2 and 3, provided that at least one is depressed mood or anhedonia, and that each symptom corresponds to a response of 2 or 3, except for symptom 9.^11^

Then, data collection was tabulated and stored in Microsoft Office Excel (2010). Descriptive statistics were used, such as measures of central tendency (simple frequency, mean, median, and minimum and maximum). The presence of a positive association between variables was investigated using odds ratio (OR) and relative risk (RR).

This survey was approved by the Research Ethics Committee of the Faculdades Integradas de Jahu, CAAE No. 79835717.5.0000.5427, opinion No. 2.467.313, in compliance with all ethical requirements for research with human beings described in Resolution No. 460/2012, regulated by the Brazil's National Health Council (Conselho Nacional de Saúde - CNS), through Brazil's Ministry of Health.^12^ The criteria proposed by the STROBE Checklist for cross-sectional studies were considered in the presentation of this study.^13^

## RESULTS

The participants in this survey included 49 professors at a university, 31 (63%) women and 18 (37%) men, teaching courses in the Humanities, Exact

Sciences, and Biological Sciences. Their ages ranged from 29-78, predominantly in the 40-49 age group, with 34.69% of the participants. Table 1 presents the general results regarding social and demographic data, lifestyle and health habits, and screening for mental disorders.

**Table 1:** Social and demographic results, health information, screening for mental disorders, and use of medication by university professors in the inland of São Paulo, Brazil, in 2021.

Social, demographic, and occupational information: a cross-sectional study	n	%
Variables		
Age group (years)		
20-29	2	4.8
30-39	16	32.6
40-49	17	34.6
50-59	8	16.3
60+	6	12.2
Length of time teaching in universities (years)		
Less than 1	3	6.1
1-5	12	24.5
5+	34	69.4
Length of time at this university (years)		
Less than 1	8	16.3
1-5	12	24.5
5+	29	59.2
Subjects taught		
Business Administration	13	26.5
Biomedical Science	2	4.0
Accounting	10	20.4
Law	5	10.2
Physical Education	4	8.1
Nursing	18	36.7
Pharmaceutical Sciences	5	10.2
Languages	2	4.0
Legal Methods	1	2.0
Pedagogy	7	14.2
Graduate Studies	2	4.0
Psychology	7	14.2
Advertising	2	4.0
Weekly teaching load (hours)		
Up to 8	34	69.4
9-16	9	18.4
17+	6	12.2
Do you work elsewhere?		
Yes	42	85.7
No	7	14.3
Health information		
Smoking		
Yes	1	2.0
No	48	98.0
Have you consumed alcohol in the last 30 days?		
Yes	31	63.3
No	18	36.7
If yes, how often?		
3-4 times a week	6	12.2
Up to once a week	21	42.9
Up to once a month	3	6.1
BMI		
19-24,99	19	38.8
25-29,99	18	36.7
30-34,99	9	18.4
35-39,99	3	6.1
40+	0	0
Do you consider yourself a sedentary person?		
Yes	20	40.8
No	29	59.2
How often do you do physical exercise in a week?		
Never	11	22.4
Once or twice	20	40.8
Three times or more	17	34.7
Do you have a chronic illness?		
Yes	20	40.8
No	29	59.2
Current chronic diseases		
Autoimmune disorder	1	2.0
Cardiovascular disorder	6	12.2
Endocrine/metabolic disorder	5	10.2
Mental disorder	8	16.3
Other	2	4.0
Continued-use medication		
Yes	32	65.3
No	17	34.7
Medications according to ATC Classification		
C - Cardiovascular system	14	28.6
R - Respiratory system	2	4.0
A - Alimentary tract and metabolism	4	8.2
N - Nervous system	9	18.4
G - Genito urinary system and sex hormones	1	2.0
H - Systemic hormonal preparations, excluding sex hormones and insulins	3	6.1
B - Blood and blood-forming organs	1	2.0
Score suggesting CMD - SRQ-20		
Women	11	35.5
Men	3	16.7
Total	14	28.6
Score suggesting depressive episode - PHQ-9 - Continuous variable		
Minimal depression	26	53.1
Mild depression	10	20.4
Moderate depression	7	14.3
Moderately severe depression	5	10.2
Severe depression	1	2
At least five symptoms with scores of 2 or 3	14	28.5

ATC = Anatomical Therapeutic Chemical; BMI = body mass index; PHQ-9 = Patient Health Questionnaire; SRQ = Self Reporting Questionnaire; CMD = common mental disorder.

### PATIENT HEALTH QUESTIONNAIRE

Most participants, 69.4% (34), work up to 8 hours a week as professors, and 85.7% (42) also have another occupation.

Among their health information, only one participant (2%) reported smoking; however, 63.3% (31) reported drinking alcohol in the last month, 12.2% (6) having done so at least three times a week.

The body mass index (BMI) of 61.2% (30) of the participants was above the recommended level, and 40.8% (20) reported being sedentary. A chronic illness was reported by 40.8% (20) of the participants; however, 65.3% (32) reported continued-use medication.

Continued-use medications were classified according to the Anatomical Therapeutic Chemical Classification, a method the World Health Organization (WHO) uses to classify therapeutically active substances according to the target organ or system and their chemical-pharmacological properties.^14^ They were classified within their first level, and seven out of 14 classes were reported. It is worth noting that the group "C" was the most commonly used, with 28.6% (14) of participants using it for cardiovascular conditions, and group "N" with 18.4% (9) for nervous system/psychoactive medications.

Participants with CMD were 28.5% (14). The continuous variable, with the sum of the answers, found moderate or greater depression in 26.5% (13) of the participants; considering at least five symptoms with answers 2-3, 28.5% (14) of the participants had depression.

In the analysis of risk factors (RR), considering CMD, a positive association was found between sedentary lifestyle, being overweight or obese, drinking alcohol at least once a week, and having worked as a professor for less than 5 years (Table 2).

**Table 2:** Relative risk of the variables physical activity, body mass index, alcohol use and length of time teaching for the outcome common mental disorder, São Paulo, Brazil, 2021

Risk factors	With outcome	No outcome	Total	RR
Sedentary lifestyle				
Yes	7	4	11	3.5
No	7	31	38	
Overweight or obese				
Yes	10	20	30	1.6
No	4	15	19	
Drinking alcohol at least once a week				
Yes	10	17	27	1.7
No	4	18	22	
Teaching for less than 5 years				
Yes	8	7	15	3.1
No	6	28	34	

RR = relative risk.

## DISCUSSION

A similar study with university professors in Bahia, Brazil, found a similar prevalence rate of 29.9%, with a positive statistical association with feeling worn out in relationships with students and dissatisfaction with the university where they work.^3^

The authors also discuss that teaching duties are often not limited to the university tripod of research, teaching, and further education, but rather are interspersed with it, and administrative and management tasks require attention and time. Sometimes perceived as bureaucratic and unnecessary, they increase the feeling of overload and can be a point to be discussed and shared.^3^

In a study with employees and students at a university in Spain, there was a significant prevalence of symptoms of depression (34.19%), anxiety (21.34%), and stress (28.14%) related to social isolation in March 2020. The authors also describe worse rates among students when compared to other groups of university employees.^15^

A second contributing element is the continued-use medication, which has shown an increased presence among the population, especially neurological/ psychoactive medication. Among professors at a private university in Goiás, Brazil, 40% reported high levels of stress and 42% reported current or previous use of psychoactive medication. The same study described a positive association between these two variables: stress and the use of psychoactive medication.^16^

Similarly, in a university in Rio Grande do Norte, Brazil, 52.2% of professors reported some stress and 7.5% of the participants reported using hypnotics or sedatives with no medical prescription. The same study described a positive association between work-related stress and alcohol consumption.^17^

Accordingly, among the other chronic non-communicable diseases (CNCDs), understanding that the combined existence of the various conditions contributes to a worsening of the health condition, habits involving an increase in cardiovascular risk have been shown to be relevant in this population. Risk factors such as a sedentary lifestyle (44%), increased BMI (72%), and increased abdominal circumference (89%) are reported among professors at a university in the inland of Bahia.^18^

When it comes to CNCDs in the teaching population, it is in line with the fact that this profession is largely performed by older people, whose lifestyle habits are compounded by genetic factors and occupational overload throughout the academic day, which leads to the need for greater care for physical, social, and mental well-being. It is also worth mentioning that women experience hormonal changes with the onset of the climacteric, which leads to an increase in cardiovascular risk and emotional burden, and that women are generally predominant among professors.^18,19^

It should be noted that this study has limitations related to the participation of a small number of professionals and its design, preventing it from establishing cause and effect relationships. The results presented encourage further research in this field, which could provide a basis for actions to plan care and monitoring of teachers' health.

## CONCLUSIONS

This study included university professors at a private university and showed that 28.5% of the participants had both CMD and depression.

We also observed an increased RR and a greater chance of developing the disorders (OR) among professors deemed to be sedentary, overweight or obese, drinking alcohol at least once a week, and having worked as a professor for less than 5 years.

This population often experiences emotional distress in addition to other CNCDs, which leads to poorer health and well-being, impacting on overall quality of life and professional performance.

We need to consider strategies for monitoring and care throughout adulthood to promote lifestyle habits that strengthen health, particularly protecting older age groups.

This study contributes to broadening knowledge about the factors that affect professors' health and can have an effect on their well-being and professional performance.
